# Real-world evaluation of ImmuCare-PRO patient-reported outcomes in melanoma patients treated with immune checkpoint inhibitors

**DOI:** 10.1016/j.esmorw.2024.100090

**Published:** 2024-11-19

**Authors:** S. Belkaïd, S. Milley, R. Saux, M. Bonjour, A. Augros, P.-J. Souquet, D. Maillet, D. Maucort-Boulch, C. Dolla, L. Thomas, S. Dalle

**Affiliations:** 1 Hospices Civils de Lyon, ImmuCare, Hôpital Lyon Sud, Service de Dermatologie, Oullins-Pierre-Bénite, France; 2 Faculté de Médecine, Universit é Claude Bernard Lyon 1, Villeurbanne, France; 3 Hospices Civils de Lyon, Pôle Santé Publique, Service de Biostatistique et Bioinformatique, Lyon, France; 4 CNRS, UMR 5558, Laboratoire de Biométrie et Biologie Évolutive, Équipe Biostatistique-Santé, Villeurbanne, France; 5 Hospices Civils de Lyon, ImmuCare, Hôpital Lyon Sud, Service de Pneumologie, Oullins-Pierre-Bénite, France; 6 Hospices Civils de Lyon, ImmuCare, Hôpital Lyon Sud, Service d'Oncologie Médicale, Oullins-Pierre-Bénite, France; 7 Hospices Civils de Lyon, Direction des Services Numériques, Lyon, France; 8 CNRS 5286, INSERM U1052, Centre de Recherche en Cancérologie de Lyon, Lyon, France

**Keywords:** immune checkpoint inhibitors, patient-reported outcome measures, melanoma, risk management, surveys and questionnaires, remote patient monitoring

## Abstract

**Background:**

Toxicity profile of immune checkpoint inhibitors (ICI) poses challenges for early detection of immune-related adverse events (IrAEs). In oncology, patient-reported outcomes (PROs) are reported to have a beneficial effect; however, their efficacy for IrAE detection in melanoma patients remains unclear. A remote patient-monitoring system was created in our department; we investigated its real-world impact in detecting grade 2 or above IrAEs occurring during ICI treatment in melanoma patients.

**Patients and methods:**

Patients receiving ICI for a melanoma were followed using a weekly online questionnaire containing 11 symptoms suggestive of IrAE. Moderate/severe symptoms generated an alert score and an intervention by an oncology nurse or physician. The system’s performance in detecting grade 2 or above IrAEs, as well as reasons for missed detections, were retrospectively assessed.

**Results:**

A total of 5202 questionnaires completed by 136 patients led to 783 (15.0%) alert scores; 64 of them were associated with 69 grade 2 or above IrAEs, with 22 (34.4%) questionnaires correctly detecting 27 grade 2 or above IrAEs, saving a mean 4.1 days on the next scheduled visit and leading to only one emergency room visit. Forty-two grade 2 or above IrAEs (mainly blood disorders, *n* = 31) were not detected. False alerts often resulted from functional or non-specific symptoms (32.3%), such as fatigue or general pain.

**Conclusion:**

The ImmuCare-PRO system correctly detected a third of moderate-to-severe IrAEs, and most of those had clinical impact such as skin toxicities, colitis, and rheumatological IrAEs. This enables earlier management and could avoid unnecessary emergency room visits.

## Highlights

• Evaluation of a PROs program for melanoma on immunotherapy was conducted.
• The PRO detected 64.7% of moderate-to-severe adverse events with clinical impact.
• The PRO saved a mean of 4.1 days on the next scheduled visit.
• False alerts were often due to non-specific symptoms (fatigue, general pain).
• High adherence and feasibility were reported in both adjuvant and metastatic settings.

## Introduction

Immune checkpoint inhibitors (ICI) are a standard treatment for metastatic melanoma and as adjuvant therapy following high-risk melanoma resection.^1^, ^2^, ^3^, ^4^ The toxicity profile of ICI is distinct,^5^ characterized by unpredictable and occasionally delayed onset of immune-related adverse events (IrAEs). Therefore, prompt management is necessary due to their potential severity, including fatal outcomes.^6^ However, the scheduled clinical visit intervals, which range from 3 to 6 weeks, and the possibility of patients underreporting their symptoms^7^ may lead to delays and complications in diagnosing IrAE.^8^

To address this issue, patient-reported outcomes (PROs) systems have been implemented. These involve reports directly from patients, without interpretation by physicians or others,^9^ and have shown positive impacts in oncology. Patient-completed questionnaires have been used to gather data on the benefits of these remote patient monitoring (RPM) systems, including improved adverse event management,^10^^,^^11^ enhanced survival and quality of life,^12^ and reductions in hospital admissions and overall costs.^11^ While investigating the use of an RPM system based on electronic PROs (ePROs) in ICI treatments, authors such as Mendoza et al. (2018), Tolstrup et al. (2019), and Zhang et al. (2022) have suggested tailoring symptom selection using the PRO version of the Common Terminology Criteria for Adverse Events (PRO-CTCAE) to detect IrAEs.^13^, ^14^, ^15^ Despite its feasibility, the efficacy of such a tailored RPM system in detecting IrAEs in melanoma patients remains uncertain.

In their randomized controlled trial (RCT), Tolstrup et al. (2020) found no significant difference in grade 3 or 4 IrAEs among melanoma patients who used a RPM system tailored with PRO-CTCAE.^16^ This may be explained by the absence of early intervention in case of alert, as was the case in the RCT conducted by Zhang et al. (2022).^13^ Their trial reported a reduction in grade 3 or 4 IrAEs and emergency room visits among ICI users, but did not include patients with melanoma.^13^ To address these gaps, our department developed ImmuCare-PRO in 2018. For this we tailored items inspired by PRO-CTCAE and incorporated systematic alerts with earlier appointment options. After several years of use, we conducted a real-world study to investigate the impact of detecting grade 2 or higher IrAEs during ICI treatment in melanoma patients.

## Patients and methods

### Setting and eligibility criteria

We conducted a single-center real-world historical (retrospective) cohort study in our dermatology department at Hôpital Lyon Sud, France, specializing in advanced and metastatic melanoma treatment. The study included adults undergoing ICI treatment for adjuvant or metastatic melanoma indications, enrolled in the RPM system before 1 January 2022, and having completed at least one questionnaire. Patients with medical, social, or psychiatric conditions that could affect informed consent or patient uncomfortable with Internet tools or the French language were excluded. An oncology nurse addressed these issues through weekly calls. Data from ePRO questionnaires administered between 23 March 2018 and 1 August 2022 were analyzed.

### ImmuCare-PRO RPM system

The ImmuCare-PRO RPM system comprises weekly online ePRO questionnaires that assess symptoms suggestive of IrAEs. At first ICI treatment, patients meet the dedicated oncology nurse and receive information about the RPM system. If patients agree to take part in this, they are registered on the ‘myHCL’ website, a personal and secure patient portal. Then, every week, patients receive a reminder by text message or e-mail to complete the questionnaire. The questionnaire can be completed on the ‘myHCL’ website or through a dedicated smartphone application (myHCL, available for Android and iOS). In the absence of a reply after 7 days, patients received a new reminder by text message or e-mail. Patients may complete additional questionnaires if desired. Participation continues throughout ICI treatment and ceases only if treatment is discontinued.

**Figure 1:**
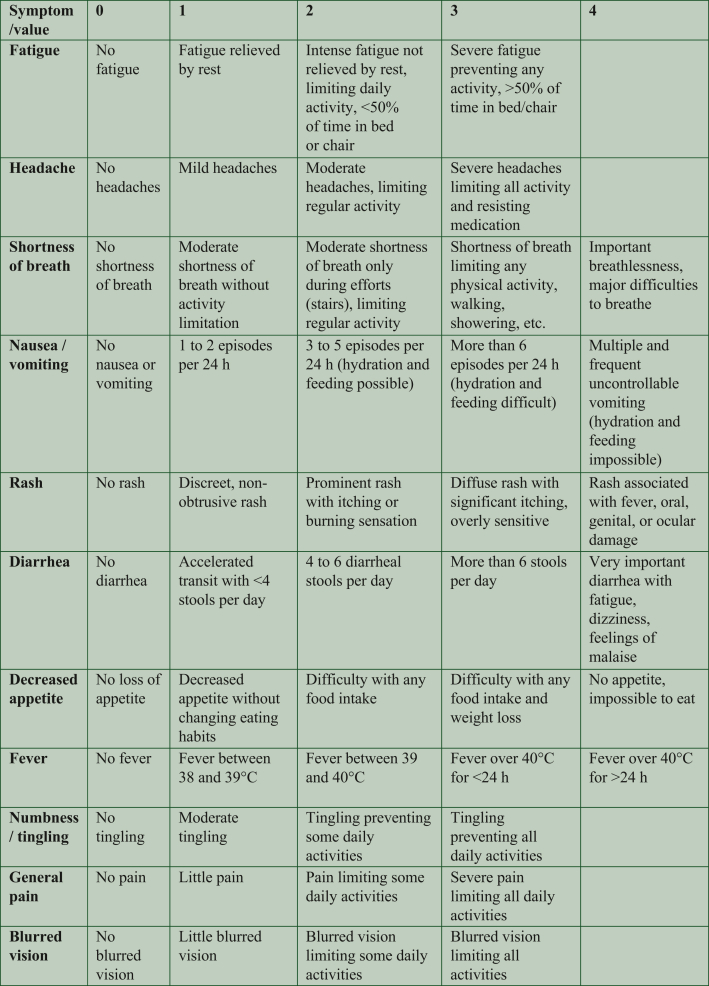
**Variables collected by the ImmuCare-PRO questionnaire.** Of note, at the end of the questionnaire there is a free-text field preceded by ‘Is there anything else you’d like us to know, or to call you back?’ The original questionnaire is in French (Supplementary Figure S1, available at https://doi.org/10.1016/j.esmorw.2024.100090).

**Figure 2:**
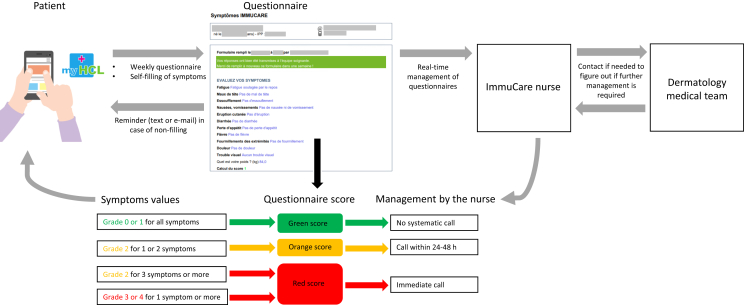
**How the ImmuCare-PRO remote patient monitoring system works**.

The *ad hoc* questionnaire, developed in French language, contains questions regarding fever, as defined by the CTCAE version 5.0 (CTCAE v5) and 10 other symptoms inspired by items of the PRO-CTCAE: fatigue, headache, shortness of breath, nausea/vomiting, rash, diarrhea, decreased appetite, numbness/tingling, general pain, and blurred vision. This 11-item questionnaire was validated by a working group comprising physicians specialized in the care of patients treated with ICI (medical oncologists, dermatologists, pneumologists, and other organ specialists) and the Hospices Civils de Lyon Information and Innovation Systems Department. Patients choose a single answer for each symptom from four (or five) options, corresponding to grades ranging from 0 to 3 (or 4), as inspired by the CTCAE v5 and PRO-CTCAE. Nausea/vomiting were merged in a single item for simplicity, and severity was measured by the number of vomiting episodes and/or the possibility of hydration/feeding, since the CTCAE grading system for the vomiting item is not well suited to an ePRO (Figure 1, original questionnaire in French Supplementary Figure S1, available at https://doi.org/10.1016/j.esmorw.2024.100090). The symptoms are then classified as absent/slight (grade 0/1), moderate (grade 2), or severe (grade 3 or 4). Patients may also provide additional comments in a free-text field (‘Is there anything else you’d like us to know, or to call you back?’), such as requesting a call, even in the absence of symptoms or not to be called back in case of stable and chronic symptoms. The questionnaires are automatically recorded in the electronic health record (EHR), and an algorithm converts the patient’s answers into a score of green, orange, or red, based on the severity of the registered symptoms. The score is green if all symptoms are grade 0 or 1; orange if one or two symptoms are grade 2; red if three symptoms (or more) are of grade 2 or if at least one symptom is grade 3 or 4. The dedicated oncology nurse receives all questionnaires in real-time through online software, which displays the current score (green, orange, or red) and the history of the three previous scores. An alert is triggered for orange and red scores. Alerts are taken into account throughout working hours (9:00 am to 4:30 pm). The oncology nurse is required to call the patient within 24-48 h for an orange score and immediately for a red score. Despite the establishment of these timeframes, they are not subjected to rigorous enforcement at the pathway level nor to close monitoring. Patients also receive the score of their questionnaire on the ‘myHCL’ portal, and are informed that they will be called back if it is orange or red. If the situation requires medical intervention, the nurse refers the patient to their physician to determine whether additional medical management is necessary (Figure 2). Even in the absence of an alert, the oncology nurse systematically calls each patient once a month to maintain human interaction in the monitoring process and ensure that symptoms are not underestimated.

### Data collection

Retrospective data were collected from each EHR, including completed ePRO and IrAE (graded according to CTCAE v5) diagnosed during the ImmuCare-PRO RPM system. Demographic data such as sex, age, and socio-occupational status, as well as melanoma characteristics and ICI treatment modalities were also collected. The alert score was collected for each case. We investigated whether there was an association between the occurrence of grade 2 or higher IrAE and the time from the alert-generating questionnaire to the diagnosis of IrAE, compared to the time to the next scheduled hospital visit. An IrAE was considered correctly detected if it was preceded by a questionnaire with an alert score and related symptoms. To avoid counting the same IrAE multiple times, only the first questionnaire was considered if several successive alert score questionnaires were related to the same IrAE. The analysis was focused on clinically relevant grade 2 or higher IrAEs, as these may require prompt management.

### Objectives

The primary objective was to determine the proportion of correctly detected CTCAE v5 grade 2 or above all IrAEs by the ImmuCare-PRO RPM system among all grade 2 or above IrAEs (clinical and blood tests-related) experienced by patients. The secondary objectives were to determine what follow-up was given after an alert score, the time from IrAEs correct detection by the RPM and next scheduled visit, the proportion and characteristics of grade 2 or above IrAEs with no prior alert score, and the proportion and characteristics of alert scores without any associated grade 2 or above IrAEs.

### Regulatory aspects and statistical analyses

The study was approved by the Scientific and Ethics Committee of Hospices Civils de Lyon (n°22_424) and adhered to French data protection guidelines set by the Commission Nationale de l'Informatique et des Libertés. No financial compensation was provided.

Statistical analyses utilized descriptive and inferential methods, with significance set at *P* < 0.05. Differences between groups were tested using the Wilcoxon or Kruskal–Wallis test for continuous variables and Fisher’s exact test for categorical variables. Analysis of factors associated with the genesis of red or orange scores was carried out using a univariate mixed logistic regression model. It considered sex, the presence of cerebral metastasis, ICI indication, and therapeutic regimen [ICI combination or single-agent anti- programmed cell death protein 1 (PD-1) therapy] as fixed effect, and the patient as random effect. The effect of factors on the increase of occurrence of alert scores was quantified through the adjusted odds ratio (aOR) with the associated 95% confidence interval (95% CI). For performance metrics [sensitivity (Se), specificity (Sp), positive predictive value (PPV), negative predictive value (NPV)], we considered as true positive a correctly detected grade 2 or above IrAE; as true negative a green questionnaire followed by the absence of grade 2 or above IrAE; as false positive an alert score unrelated to a grade 2 or above IrAE; and as false negative a grade 2 or above IrAE which were not correctly detected by the RPM system.

## Results

### Baseline patient’s demographics and disease characteristics

From 28 March 2018 to 1 January 2022, 139 patients starting a new ICI treatment were registered in the ImmuCare-PRO RPM system, and no patient refused to participate in the study. After first registration, three patients did not complete any questionnaire and were not included in the study. A total of 136 patients (55.9% male and 44.1% female) were included for the analysis (Supplementary Figure S2, available at https://doi.org/10.1016/j.esmorw.2024.100090). The median age of the patients was 57.5 years. Of these patients, 90 (66.2%) received adjuvant ICI and 46 (33.8%) received ICI for a metastatic melanoma: 13 (28.3%) received a combination of nivolumab and ipilimumab and 33 (71.7%) a single anti-PD-1 agent (Table 1).

**Table 1:** Baseline patient demographics and disease characteristics

	Total population *n* = 136
Male sex, *n* (%)	76 (55.9)
Median age, years (range)	57.5 (23-85)
Socio-occupational status, *n* (%)	
Unemployed	4 (2.9)
Employee, laborer, farmer	50 (36.8)
Craftsman, tradesman	8 (5.8)
Middle and higher intellectual professions	42 (30.9)
Missing data	32 (23.6)
Breslow index in millimeters, median (range)	2.7 (0.3-21.0)
Lactate dehydrogenase in IU/l, median (range)	196.5 (130.0-790.0)
ECOG performance status 0, *n* (%)	124 (91.2)
Indication of ICI treatment, *n* (%)	
Adjuvant indication	90 (66.2)
Metastatic indication	46 (33.8)
ICI, *n* (%)	
Ipilimumab + nivolumab	13 (9.6)
Nivolumab alone	80 (58.8)
Pembrolizumab alone	43 (31.6)
Duration of ICI treatment in weeks, median (range)	48.1 (1.0-237.3)
Earlier line of treatment, *n* (%)	16 (11.8)
Adjuvant indication, *n* (%)	*n* = 90
Positive SLNB	44 (48.9)
Macroscopic lymph node involvement	30 (33.3)
Visceral involvement	16 (17.8)^a^
Metastatic sites, *n* (%)	*n* = 46
Lymph node	33 (71.7)
Lung	20 (43.5)
Liver	14 (30.4)
Other digestive site	10 (21.7)
Central nervous system and brain	14 (30.4)
Bone	10 (21.7)
Skin	18 (39.1)
Other	4 (8.7)
Continuation of ImmuCare-PRO RPM system at the end of the data collection, *n* (%)	
Continuation	32 (23.5)
Discontinuation	104 (76.5)
Scheduled interruption of treatment	51/104 (49.0)
Disease aggravation and change of treatment	44/104 (42.3)
Continuation of treatment in a different center	3/104 (2.9)
Death	3/104 (2.9)
Refusal to continue, non-compliance	3/104 (2.9)

ECOG, Eastern Cooperative Oncology Group; ICI, immune checkpoint inhibitor; IU, international units; RPM, remote patient monitoring; SLNB, sentinel lymph node biopsy.

a Including two cerebral metastases.

### Description of ImmuCare-PRO

We analyzed a total of 5202 questionnaires completed between 23 March 2018 and 1 August 2022, with a median follow-up of 46 weeks. The participants completed a median of 39 questionnaires (Table 2). The median adherence rate did not significantly differ according to age (*P* = 0.727), sex (*P* = 0.489), socio-occupational status (*P* = 0.605), ICI indication (*P* = 0.107), or presence of brain metastases (*P* = 0.115; Supplementary Table S1, available at https://doi.org/10.1016/j.esmorw.2024.100090). A total of 104 (76.5%) patients discontinued the ImmuCare-PRO: 51 (49.0%) for scheduled interruption of treatment (after 12 months of adjuvant treatment), 44 (42.3%) for a disease progression, 3 (2.9%) since treatment was continued in a different center, and 3 (2.9%) because of death. Three (2.9%) patients refused to continue, after 99, 6, and 1 questionnaires, respectively (Table 1).

**Table 2:** Description of the ImmuCare-PRO RPM system

	Total questionnaires *n* = 5202
Questionnaires, median (range)	
Follow-up time in weeks	46 (1-227)
Questionnaires per patient	39 (1-110)
Green score questionnaire per patient	31 (0-107)
Orange score questionnaire per patient	2 (0-71)
Red score questionnaire per patient	0 (0-22)
Completed questionnaires per week	0.89 (0.22-3.00)
Overall score, *n* (%)	
Green	4394 (84.5)
Orange	661 (12.7)
Red	122 (2.3)
Orange/red score related to a previously diagnosed IrAE	25 (0.5)
Questionnaires with following grade 2 or higher IrAE, *n* (%)	*n* = 64^a^
Grade 2 symptoms	51 (79.7)
Grade 3 or 4 symptoms	13 (20.3)
Questionnaires that correctly detected IrAE	22 (34.4)
Questionnaires that not correctly detected IrAE	42 (65.6)
Symptom collection for alert scores, *n* (%)	*n* = 783^b^
Fatigue	359 (45.8)
General pain	237 (30.3)
Shortness of breath	128 (16.3)
Rash	118 (15.1)
Decreased appetite	80 (10.2)
Numbness/tingling	61 (7.8)
Headache	60 (7.7)
Nausea/vomiting	50 (6.4)
Diarrhea	45 (5.7)
Blurred vision	42 (5.4)
Fever	40 (5.1)
Management after an alert score, *n* (%)	*n* = 783^b^
By the oncology nurse independently	745 (95.1)
Teleconsultation	19 (2.4)
Rapid hospital admission	15 (1.9)
General practitioner visit	1 (0.1)
Emergency room visit	3 (0.4)
Time between questionnaire and consultation or hospital admission in days, mean (SD)	1.5 (2.3)

IrAE, immune-related adverse event; RPM, remote patient monitoring; SD, standard deviation.

a Sixty-four questionnaires were associated to 69 grade 2 or higher IrAEs in 50 patients.

b After exclusion of the 25 orange/red scores associated with an IrAE that has been already described.

Among the 5202 questionnaires, the score was green for 4394 (84.5%), orange for 661 (12.7%), and red for 122 (2.3%), with an overall alert rate of 15.0%. Twenty-five (0.5%) questionnaires with an orange or red score were associated with an IrAE that had been previously described and were not included in the analysis as specified above. The most frequently reported symptom was fatigue (*n* = 359, 45.8%), followed by general pain (*n* = 237, 30.3%), shortness of breath (*n* = 128, 16.3%), and rash (*n* = 118, 15.1%).

### Adverse events

Out of the 136 patients included in the study, 50 patients (36.8%) experienced 69 grade 2 or higher IrAEs (Table 3). Among them, 12 (8.8%) patients experienced at least one grade 3 or 4 IrAEs. Specifically, 4 out of 13 patients treated with ipilimumab plus nivolumab (30.8%) and 8 out of 123 patients treated with anti-PD-1 alone (6.5%) experienced grade 3 or 4 IrAEs. Thirty-one grade 2 or higher IrAEs (44.9%) were blood disorders without clinical impact, such as hypophysitis, thyroiditis, and nephritis. Blood test disorders which manifested with symptoms (such as fatigue or vomiting) were considered as symptomatic adverse events. After excluding blood disorder IrAEs without clinical impact, 34 questionnaires were found to be associated with 38 grade 2 or higher IrAEs. Supplementary Figure S3, available at https://doi.org/10.1016/j.esmorw.2024.100090, shows the distribution of green, orange, and red questionnaires, as well as the occurrence of IrAEs over time.

**Table 3:** Characteristics of grade 2 or higher IrAE

Patients with at least one grade 2 or higher IrAE	*n* = 50^a^	
	With at least one correctly detected IrAE	With at least one not correctly detected IrAE
	*n* = 20^b^	*n* = 38^b^
Median age, years (range)	59 (29-84)	50 (23-83)
Male, *n* (%)	9 (45)	22 (57.9)
Metastatic melanoma, *n* (%)	11 (55)	17 (44.7)
Single-agent anti-PD-1, *n* (%)	15 (75)	32 (84.2)

**Grade 2 or higher IrAE**	***n* = 69**	

	Correctly detected by an alert score	Not correctly detected
*n* = 27^c^	*n* = 42
CTCAE v5 grade 3 or 4 IrAEs, *n* (%)	10 (37.0)	7 (16.7)
Thyroiditis, *n* (%)	3 (11.1)	16 (38.0)
Colitis and digestive IrAEs, *n* (%)	4 (14.8)	5 (11.9)
Hepatitis, *n* (%)	3 (11.1)	5 (11.9)
Skin toxicity, *n* (%)	7 (25.9)	1 (2.4)
Hypophysitis, *n* (%)	2 (7.4)	4 (9.5)
Hyperlipasemia, *n* (%)	0 (0)	5 (11.9)
Rheumatological IrAEs, *n* (%)	4 (14.8)	0 (0)
Dry syndrome, *n* (%)	1 (3.7)	2 (4.8)
Myositis, *n* (%)	1 (3.7)	1 (2.4)
Ophthalmological toxicity, *n* (%)	1 (3.7)	1 (2.4)
Immunoallergic interstitial nephritis, *n* (%)	0 (0)	1 (2.4)
Mixed connective tissue disease, *n* (%)	1 (3.7)	0 (0)
Interstitial pneumonitis, *n* (%)	0 (0)	1 (2.4)
Interval between alert score and diagnosis of IrAE, median (range)	3.0 (0.0-25.0)	
Days saved compared to next scheduled visit, mean (range)	4.1 (0.0-20.0)	
IrAE preceded by unrelated orange/red score, *n* (%)		7 (16.7)
Rapid hospital admission related to unreported IrAEs, *n* (%)		7 (16.7)
Reasons for not reporting IrAE, *n* (%)		
Blood disorders IrAEs without clinical impact		31 (73.8)
IrAE-related symptoms not collected by the questionnaire		2 (4.8)
Absence of completion of questionnaire		1 (2.4)
Absence of mention of the IrAE-related symptoms		8 (19.0)

CTCAE v5, Criteria for Adverse Events Version 5.0; ICI, immune checkpoint inhibitor; IrAE, immune-related adverse event; PD-1, programmed cell death protein 1; SD, standard deviation.

a Two patients presented two simultaneous toxicities and one patient four simultaneous toxicities.

b Eight patients had both reported and unreported IrAEs.

c Three patients developed several simultaneous grade 2 or higher IrAEs. Two patients developed two successive grade 2 or higher IrAEs at different times of the follow-up.

### Alert scores related to grade 2 or higher IrAEs

Of the 64 questionnaires associated with grade 2 or higher IrAEs, 22 (34.4%) were alert scores that correctly detected the 27 IrAEs among 20 patients (40% of patients with IrAEs, Supplementary Table S2, available at https://doi.org/10.1016/j.esmorw.2024.100090), mainly skin toxicities (*n* = 7). These IrAEs were grade 2 (*n* = 17, 63%) or grade 3 or 4 (*n* = 10, 37%; Table 3). After excluding blood disorders without clinical impact and considering only questionnaires associated with grade 2 or higher IrAEs, 22 out of 34 alert scores (64.7%) correctly detected an IrAE. When considering all grade 2 or above IrAEs, the Se of ImmuCare-PRO was 39.1% (95% CI 28.5% to 50.91%), Sp was 85.1% (95% CI 84.1% to 86.1%), PPV was 3.4% (95% CI 2.7% to 4.9%), and NPV was 99.0% (95% CI 98.7% to 99.3%). When considering only symptomatic AEs, Se was 71.1% (95% CI 55.2% to 83.0%) Sp was 85.2% (95% CI 0.84% to 0.86%), PPV was 3.4% (95% CI 2.4% to 4.9%), and NPV was 99.8% (95% CI 99.5% to 99.9%).

Regarding the management of the 22 alert scores, 13 (59.1%) were independently managed by the oncology nurse, while 9 (40.9%) required additional medical evaluation. Of these, two were managed through teleconsultation, two through the next scheduled visit (for grade 2 IrAEs), four through rapid hospital admission (grade 3), and one through an unscheduled hospital admission via the emergency room (Supplementary Table S3, available at https://doi.org/10.1016/j.esmorw.2024.100090). The median time between an alert score and the diagnosis of the corresponding IrAEs was 3.0 days. This resulted in a mean time savings of 4.1 days on the next scheduled visit. The maximum number of days saved was 20 days for one patient (Table 3).

### Grade 2 or higher IrAEs with no prior alert score

Among the 69 grade 2 or higher IrAEs, 42 (60.9%) were not detected by an alert score in 38 patients, mainly thyroiditis (*n* = 16). These IrAEs were grade 2 (*n* = 35, 83.3%) and grade 3 or 4 (*n* = 7, 16.7%), and led to seven unscheduled hospital admissions. Out of all 4394 green score questionnaires, only 35 (0.8%) were followed by grade 2 or higher IrAEs. Additionally, seven IrAEs were preceded by an orange/red score, but the symptoms reported were not related to the observed IrAEs (Table 3).

Thirty-one (73.8%) IrAEs were blood disorders without clinical impact and were not detected by ImmuCare-PRO. Two (4.8%) IrAEs were not detected because the IrAE-related symptoms were not collected by the ImmuCare-PRO questionnaire (two patients with dry mouth syndrome, but without blurred vision). One (2.4%) IrAE consisted of a colitis and was not detected because the patient did not complete the questionnaire at the time the symptoms occurred. Eight patients (19.0%) completed the questionnaire but did not report any IrAE-related symptoms, even though the questionnaire included these symptoms, which generated an incorrect green score (Table 3).

### Alert scores without any associated grade 2 or higher IrAE

Among 5202 questionnaires, 761 (14.6%) were not associated with a grade 2 or higher IrAE: 641 (84.2%) orange score and 120 (15.8%) red score. The main symptoms reported by these patients were fatigue (*n* = 348, 45.7%), general pain (*n* = 231, 30.3%), and shortness of breath (*n* = 128, 16.8%). Among these 761 alert scores, 246 (32.3%) were due to functional or non-specific symptoms (especially fatigue); 142 (18.7%) to a previous medical condition (e.g. general pain in a patient with known degenerative osteoarthritis); 140 (18.4%) to a simultaneous independent medical condition (e.g. grade 3 diarrhea in a patient with viral gastroenteritis); 111 (14.6%) to unintentional patient input error; 63 (8.3%) reported overestimated symptoms of grade 1 IrAEs (e.g. skin toxicity); and 59 (7.8%) to symptoms related to tumor burden itself (Supplementary Table S4, available at https://doi.org/10.1016/j.esmorw.2024.100090).

No significant association was found between the occurrence of an alert score without any related grade 2 or higher IrAE and sex, age, presence of cerebral metastases, or number of questionnaires per week. However, receiving an ICI combination was significantly associated with an alert score without a grade 2 or higher IrAE compared to single-agent anti-PD-1 [aOR 5.47; 95% CI 1.51-21.16; Supplementary Table S5, available at https://doi.org/10.1016/j.esmorw.2024.100090].

## Discussion

In the present study, the ImmuCare-PRO RPM system correctly detected one-third of moderate-to-severe (grade 2 or higher) IrAEs. These IrAEs were mainly skin toxicities and colitis, which are the two main toxicities of ICI.^17^ RPM systems based on ePRO are designed to detect only symptomatic IrAEs; in this study most symptomatic IrAEs were correctly detected, leading to an earlier management of the IrAEs. Moreover, some blood test disorders, such as thyroiditis, hypophysitis, or cytolysis, may also be symptomatic (e.g. fatigue, vomiting, pain, nausea/vomiting), and therefore detected by the RPM system. Most grade 2 or higher IrAEs that did not trigger an alert were for a blood disorder without clinical impact, underlining that the RPM system should complement regular blood test monitoring, and was integrated as such within our center. Early detection is crucial for immune-related colitis, as it may lead to serious complications (perforation, sepsis, or even death) if managed late^18^ but also for rheumatological and skin toxicities as their early management allows for an improvement in the quality of life of patients.^17^ Also, only one patient required a visit to the emergency room, which may also be beneficial for the patient’s quality of life. This is consistent with the results reported by Zhang et al. who observed a reduction in emergency room visits in patients followed by their RPM system based on ePRO versus standard care [hazard ratio 0.46 (95% CI 0.26-0.81)].^13^ Finally, the NPV of the RPM system was close to 100%, indicating that the absence of alert is a good reflection of the absence of IrAE.

The ImmuCare-PRO questionnaire adequately covered clinical IrAEs, but false alerts were frequent and often related to functional and non-specific symptoms, mostly fatigue and general pain. Msaouel et al.^19^ reported similar findings, noting that these symptoms are often chronic and multifactorial. To improve the specificity of the alert system, we suggest replacing the generic term ‘general pain’ with ‘joint pain’ (an item included in the PRO-CTCAE library) to better screen for immune-related arthralgia. Furthermore, nearly a fifth of these false alerts were related to a concomitant medical condition, making it difficult for patients to distinguish between an intercurrent disease and an authentic IrAE. However, most of these situations could be managed by the oncology nurse alone. To enhance the relevance of symptoms, we suggest using the baseline status as a reference for symptom evolution, as proposed by Basch et al.^20^ and da Silva Lopes et al.^21^ This approach can better distinguish symptoms related to IrAEs from those related to tumor burden in metastatic situations or previous independent comorbidities. Additionally, we observed that patients treated with a combination of ICI completed significantly more false alerts than other patients. The reason for this may be that these patients are more vigilant to severe IrAEs due to a higher proportion of expected IrAEs and more tumor symptoms, as this combination is often used in the context of more severe metastatic disease. A weighting of the alert score according to the reported symptoms could also be proposed, by attributing a red score to grade 2 symptoms such as for diarrhea in order to better detect immune-induced colitis that are potentially severe. A subsequent serial evaluation would be beneficial in determining whether these changes enhance the system’s performance. Although the ePRO items are not exclusive to melanoma nor ICI treatment, RPM systems based on ePRO remain pertinent if adapted to the target population, as recommended by the European Society for Medical Oncology.^22^ The ImmuCare-PRO questionnaire focuses indeed on the symptoms most suggestive of IrAEs, which are different from those of chemotherapy or targeted therapies. However, certain items such as fatigue or diarrhea are adapted to most tumor locations and most treatments.

Overall adherence was high in the present study, exceeding the 75% adherence rate reported in the systematic review of ICI-tailored RPM systems based on ePRO. However, adherence was defined differently in each included study^23^ and the choice to measure adherence by the number of completed questionnaires per week may have led to an overestimation, as some patients completed more than a mean of 1 questionnaire per week. We did not identify any factors affecting adherence, suggesting that ImmuCare-PRO can be used in both adjuvant and metastatic situation. The RPM system appears to be well accepted, as only three patients decided to stop the PRO follow-up. An RPM system based on ePRO makes it possible to follow up more patients than a classic telephone follow-up, which can be time-consuming. Despite this, systems based on ePROs are mainly intended for people comfortable with Internet tools, possibly excluding some patients. Even those who are comfortable with the Internet may miss the human interaction during RPM system follow-ups. Therefore, ImmuCare-PRO addresses this by having an oncology nurse make monthly calls to all patients, maintaining a human dimension to the telemonitoring. From the perspective of the oncology nurse, the RPM system is an efficient means of identifying patients for contact, with the time spent on questionnaire management relatively brief. The questionnaires provide the nurse with a foundation for discussion during calls and facilitate the nurse’s integration into the medical team’s decision-making process through the nurse’s expertise in detecting IrAEs. Additionally, patients reported that the questionnaires were relatively quick to complete and enhanced their confidence through the reassurance of having their responses read by a member of the team, with each patient receiving an acknowledgement of receipt.

The main limitation of the present study is related to its non-comparative nature, which precludes us from investigating a reduction in the occurrence of grade 3 or 4 IrAEs through early detection. A prospective randomized controlled study, comparing ImmuCare-PRO to standard care, could further explore this, along with secondary outcomes such as treatment discontinuation, survival rates, and emergency room visits. The strength of the present study is the evaluation of the ImmuCare-PRO RPM system in real-world conditions. It includes a cohort of patients treated with ICI for metastatic and adjuvant melanoma, which is relevant to the recent expanding ICI indications for stages IIB/IIC melanoma.^3^ These results could be generalized to all patients treated with ICI for melanoma.

### Conclusions

The ImmuCare-PRO RPM system correctly detected a third of moderate-to-severe IrAEs, and most of those with clinical impact such as skin toxicities, colitis, and rheumatological IrAEs. This enables earlier management and could avoid unnecessary emergency room visits.
