# Supplementary material for: Real-world evaluation of ImmuCare-PRO patient-reported outcomes in melanoma patients treated with immune checkpoint inhibitors
Source: ESMO Real World Data Digit Oncol. 2024 Nov 19;6:100090. doi: 10.1016/j.esmorw.2024.100090 (PMC12836578; doi:10.1016/j.esmorw.2024.100090)
Supplement: Supplementary Material [file mmc2.docx]

**Supplementary data**

| Age |  | p=0.727 |
| --- | --- | --- |
| Sex, median [IQR] |  | p=0.489 |
| male | 0.87 [0.64-1.00] |  |
| female | 0.90 [0.66-1.01] |  |
| Socio-occupational status, median [IQR] |  | p=0.605 |
| unemployed | 0.79 [0.69-0.88] |  |
| employee, laborer, farmer | 0.89 [0.69-1.00] |  |
| craftsman, tradesman | 0.70 [0.54-0.94] |  |
| middle and higher intellectual professions | 0.89 [0.37-3.00] |  |
| ICI indication, median [IQR] |  | p=0.107 |
| adjuvant | 0.91 [0.72-1.00] |  |
| metastatic | 0.70 [0.49-1.05] |  |
| ICI regimen treatment, median [IQR] |  | p=0.991 |
| ipilimumab plus nivolumab | 0.78 [0.50-1.10] |  |
| nivolumab or pembrolizumab alone | 0.89 [0.24-2.72] |  |
| Presence of brain metastases, median [IQR] |  | p=0.115 |
| yes | 0.66 [0.46-1.00] |  |
| no | 0.93 [0.59-1.12] |  |

**Supplementary Table S1 – Number of completed questionnaires per week, by variables.**

ICI: immune-checkpoint inhibitor, IQR: interquartile range

| **Age** | **Sex** | **ICI Indication** | **Questionnaire  color (score)** | **Reported  symptom(s)** | **Symptom grade, CTCAE v5** | **Management** | **Delay to diagnosis, in days** | **Saved time, in days** | **IrAEs type** | **IrAEs grade, CTCAE v5** |
| --- | --- | --- | --- | --- | --- | --- | --- | --- | --- | --- |
| 46 | Female | Metastatic | Orange | Rash | 2 | oncology nurse  independently | 10 | 0 | skin toxicity | 2 |
|  |  |  | Orange | Fatigue, diarrhea | 2 | rapid hospital admission | 2 | 20 | colitis/digestive IrAEs, hepatitis, hypophysitis, myositis | 3 |
| 61 | Female | Metastatic | Orange | Rash | 2 | oncology nurse  independently | 4 | 0 | skin toxicity | 2 |
| 46 | Female | Adjuvant | Orange | Blurred vision | 2 | oncology nurse  independently | 1 | 0 | dry syndrome | 2 |
| 68 | Male | Adjuvant | Red | Fatigue, nausea/vomiting,  loss of appetite | 2 | rapid hospital admission | 0 | 7 | hypophysitis | 3 |
| 29 | Male | Adjuvant | Orange | Fatigue, blurred vision | 2 | next scheduled visit at hospital | 1 | 0 | ocular toxicity | 2 |
| 71 | Female | Metastatic | Orange | Rash | 2 | rapid hospital admission | 1 | 11 | mixed connective  tissue disease | 3 |
| 38 | Male | Adjuvant | Orange | Rash | 2 | oncology nurse independently | 4 | 0 | skin toxicity | 2 |
| 81 | Male | Metastatic | Orange | Rash | 2 | next scheduled visit at hospital | 4 | 2 | skin toxicity | 3 |
| 29 | Female | Adjuvant | Orange | General Pain | 2 | hospital admission through the  emergency room | 3 | 20 | rheumatological IrAEs | 2 |
| 48 | Female | Adjuvant | Orange | Fatigue, nausea/vomiting | 2 | oncology nurse  independently | 21 | 0 | thyroiditis | 2 |
| 67 | Female | Adjuvant | Orange | Fatigue, loss of appetite | 3 | oncology nurse  independently | 3 | 0 | colitis/digestive IrAEs,  hepatitis | 3 |
| 71 | Female | Metastatic | Orange | Rash, general pain | 3 | rapid hospital admission | 2 | 0 | skin toxicity | 2 |
| 63 | Female | Adjuvant | Orange | Fatigue | 2 | oncology nurse  independently | 14 | 0 | thyroiditis | 2 |
| 54 | Male | Metastatic | Red | Fatigue, diarrhea,  numbness/tingling,  general pain | 2 | teleconsultation | 1 | 16 | colitis/digestive IrAEs | 3 |
| 42 | Male | Metastatic | Orange | Rash | 2 | teleconsultation | 3 | 15 | skin toxicity, thyroiditis | 2 |
|  |  |  | Orange | Fatigue | 2 | oncology nurse  independently | 0 | 0 | hepatitis | 2 |
| 55 | Male | Adjuvant | Orange | Fatigue, general pain | 2 | oncology nurse  independently | 0 | 0 | rheumatological IrAEs | 2 |
| 73 | Male | Metastatic | Orange | Rash | 2 | oncology nurse  independently | 6 | 0 | skin toxicity | 2 |
| 71 | Male | Metastatic | Orange | Fatigue, loss of appetite | 2 | oncology nurse  independently | 3 | 0 | colitis/digestive IrAEs | 2 |
| 57 | Female | Adjuvant | Orange | General Pain | 2 | oncology nurse  independently | 25 | 0 | rheumatological IrAEs | 2 |
| 84 | Female | Adjuvant | Orange | Fatigue, loss of appetite,  fever, general pain | 2 | oncology nurse  independently | 12 | 0 | rheumatological IrAEs | 2 |

**Supplementary Table S2 – characteristics of patients with alert scores related to CTCAE v5 grade 2 or higher IrAEs**

ICI: immune-checkpoint inhibitors, IrAE: immune-related adverse event, CTCAE v5: Criteria for Adverse Events Version 5.0

|  | Alert scores with correct detection of IrAEs |
| --- | --- |
|  | n=22 |
| Management of these alert scores, n (%) |  |
| management by the oncology nurse independently | 13 (59.1) |
| management after teleconsultation | 2 (9.1) |
| management at next scheduled visit at hospital | 2 (9.1) |
| management with rapid hospital admission | 4 (18.2) |
| management with unscheduled hospital admission through the emergency room | 1 (4.5) |
| Symptoms collection on alert scores related to CTCAE v5 grade 2 or higher IrAEs, n (%) |  |
| fatigue | 11 (50) |
| rash | 8 (36.4) |
| general pain | 6 (27.3) |
| decreased appetite | 4 (18.2) |
| nausea/vomiting | 2 (9.1) |
| diarrhea | 2 (9.1) |
| blurred vision | 2 (9.1) |
| fever | 1 (4.5) |
| numbness/tingling | 1 (4.5) |
| headache | 0 (0) |
| shortness of breath | 0 (0) |

**Supplementary Table S3 - Characteristics of alert scores that correctly detected CTCAE v5 grade 2 or higher IrAEs.**

IrAE: immune-related adverse event, CTCAE v5: Criteria for Adverse Events Version 5.0

|  | Alert scores without associated IrAEs | |
| --- | --- | --- |
|  | n = 761 | |
| Color of alerts scores without any associated IrAEs, n (%) | |  |
| orange score | 641 (84.2) | |
| red score | 120 (15.8) | |
| Symptoms collection, n (%) |  | |
| fatigue | 348 (45.7) | |
| general pain | 231 (30.3) | |
| shortness of breath | 128 (16.8) | |
| rash | 110 (14.5) | |
| decreased appetite | 76 (10.0) | |
| headache | 60 (7.9) | |
| numbness/tingling | 60 (7.9) | |
| nausea/vomiting | 48 (6.3) | |
| diarrhea | 43 (5.6) | |
| blurred vision | 40 (5.3) | |
| fever | 39 (5.1) | |
| Reasons of these alert scores, n (%) |  | |
| functional or non-specific symptoms | 246 (32.3) | |
| related to previous medical problem | 142 (18.7) | |
| independent medical problem | 140 (18.4) | |
| unintentional patient input error | 111 (14.6) | |
| overestimated symptoms of grade 1 IrAEs | 83 (8.3) | |
| related to tumor burden itself | 59 (7.8) | |

**Supplementary Table S4 - Alert scores without any associated CTCAE v5 grade 2 or higher IrAEs.**

IrAE: immune-related adverse event, CTCAE v5: Criteria for Adverse Events Version 5.0

|  | Alert scores without associated IrAEs |
| --- | --- |
|  | n = 761 |
| ICI combination (reference: single-agent ICI) | 5.47 [1.51;21.16], 0.01 |
| Female (reference: male) | 1.17 [0.53;2.6], 0.68 |
| Presence of brain metastases (reference: absence of brain metastases) | 1.25 [0.24;6.9], 0.78 |
| Age | 1.00 [0.98;1.03], 0.81 |
| Number of questionnaires per week | 1.81 [0.6;5.75], 0.28 |
| Metastatic ICI indication (reference: adjuvant ICI indication) | 1.31 [0.57;3.03], 0.52 |

**Supplementary Table S5 - Factors associated with orange/red scores without any related grade 2 or higher IrAEs.**

Data are aOR [95%CI], p value

IrAE: immune-related adverse event, 95%CI: 95% confidence interval, ICI: immune-checkpoint inhibitor, aOR: adjusted odds ratio

**Évaluez vos symptômes :**

**Fatigue**

🔿 Pas de fatigue. [0]

🔿 Fatigue soulagée par le repos. [1]

🔿 Fatigue intense non soulagée par le repos, limitant l’activité quotidienne, moins de 50 % du temps au lit ou fauteuil. [2]

🔿 Fatigue intense empêchant toute activité, plus de 50% du temps au lit/fauteuil. [3]

**Mal de tête**

🔿 Pas de mal de tête. [0]

🔿 Légers maux de tête. [1]

🔿 Maux de tête modérés, limitant l’activité régulière. [2]

🔿 Maux de tête importants limitant toute activité et résistant aux prises des médicaments. [3]

**Essoufflement**

🔿 Pas d’essoufflement. [0]

🔿 Essoufflement modéré sans limitation d’activité. [1]

🔿 Essoufflement modéré uniquement aux efforts (escaliers), limitant l’activité régulière. [2]

🔿 Essoufflement limitant toute activité physique, marche, douche etc.. [3]

🔿 Essoufflement important, difficultés majeures pour respirer. [4]

**Nausées, vomissements**

🔿 Pas de nausée ni de vomissement. [0]

🔿 1 à 2 épisodes par 24h. [1]

🔿 3 à 5 épisodes par 24h (hydratation et alimentation possible). [2]

🔿 Plus de 6 épisodes par 24 heures (hydratation et alimentation difficiles). [3]

🔿 Vomissements rapprochés et multiples incontrôlables (hydratation et alimentation impossibles). [4]

**Éruption cutanée**

🔿 Pas d’éruption. [0]

🔿 Éruption discrète, non gênante. [1]

🔿 Éruption bien visible avec démangeaisons ou sensation de cuisson. [2]

🔿 Éruption diffuse avec démangeaison importante très prurigineuse. [3]

🔿 Éruption associée à une fièvre, une atteinte buccale, génitale ou oculaire. [4]

**Diarrhée**

🔿 Pas de diarrhée. [0]

🔿 Accélération du transit avec moins de 4 selles par jour. [1]

🔿 4 à 6 selles diarrhéiques par jour. [2]

🔿 Plus de 6 selles par jour. [3]

🔿 Diarrhée très importante avec fatigue, vertige, sensations de malaise. [4]

**Perte d’appétit**

🔿 Pas de perte d’appétit. [0]

🔿 Diminution d’appétit sans changer les habitudes alimentaires. [1]

🔿 Difficulté pour toute prise d’aliment. [2]

🔿 Difficulté pour toute prise alimentaire et perte de poids. [3]

🔿 Aucun appétit, alimentation impossible. [4]

**Fièvre**

🔿 Pas de fièvre. [0]

🔿 Fièvre entre 38 et 39 °C. [1]

🔿 Fièvre entre 39 et 40°C. [2]

🔿 Fièvre supérieure à 40°C pendant moins de 24h. [3]

🔿 Fièvre supérieure à 40°C pendant plus de 24h. [4]

**Fourmillements des extrémités**

🔿 Pas de fourmillement. [0]

🔿 Fourmillements modérés. [1]

🔿 Fourmillement empêchant certaines des activités quotidiennes. [2]

🔿 Fourmillements empêchant toute activité quotidienne. [3]

**Douleur**

🔿 Pas de douleur. [0]

🔿 Douleur faible. [1]

🔿 Douleur limitant certaines activités quotidiennes. [2]

🔿 Douleurs intenses limitant toute activité quotidienne. [3]

**Trouble visuel**

🔿 Aucun trouble visuel. [0]

🔿 Trouble visuel peu gênant. [1]

🔿 Trouble visuel limitant certaines activités quotidiennes. [2]

🔿 Trouble visuel limitant toute activité. [3]

Souhaitez-vous nous signaler autre chose, être rappelé ?

Commentaire [ ]

**Supplementary Figure S1. ImmuCare-PRO questionnaire in French.**

 **Supplementary Figure S2 – Flowchart illustrating the selection process for analyzed cases.**


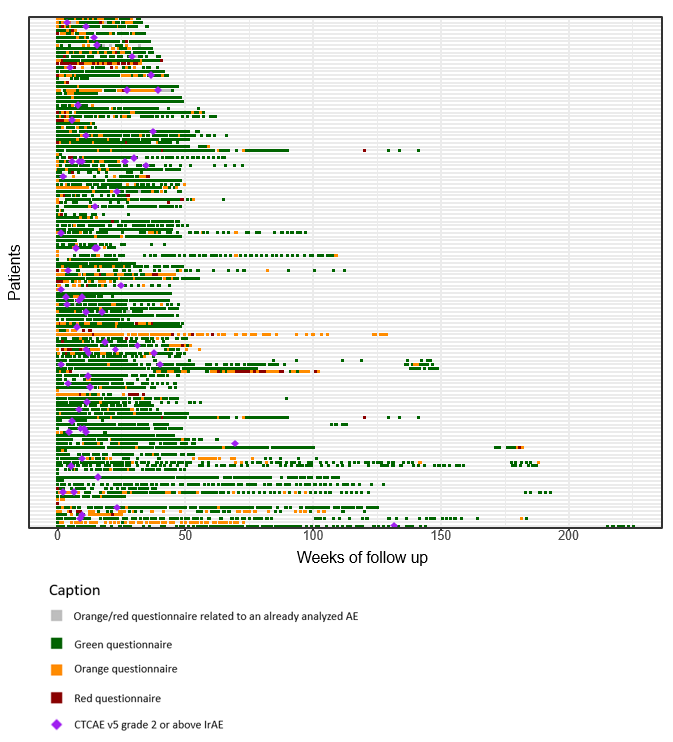
 **Supplementary Figure S3 - Distribution of green, orange, and red questionnaires, and the occurrence of IrAEs over time.**

On the y axis, the 137 patients included. On the x-axis, a visual representation of all completed questionnaires with their score (green, orange or red) and the occurrence of grade 2 or higher IrAEs as a function of time.

IrAE: immune-related adverse event
